# Esophageal Mucoepidermoid Carcinoma: A Review of 58 Cases

**DOI:** 10.3389/fonc.2022.836352

**Published:** 2022-04-13

**Authors:** Xin Wang, Yu-ping Chen, Shao-bin Chen

**Affiliations:** Department of Thoracic Surgery, Cancer Hospital of Shantou University Medical College, Shantou, China

**Keywords:** esophagus, mucoepidermoid carcinoma, surgery, treatment, prognosis

## Abstract

**Background:**

Esophageal mucoepidermoid carcinoma (EMEC) is a rare disease. The biological behavior and treatment of this malignancy are not well established.

**Methods:**

Data from 58 patients with EMEC who underwent esophagectomy were retrospectively analyzed and compared with 5028 patients with esophageal squamous cell carcinoma (ESCC). Kaplan–Meier and multivariate Cox regression analyses were conducted to investigate the association between clinicopathological factors and survival.

**Results:**

The study cohort included 36 males and 22 females with a median age of 59 years (range, 40-78 years). Of the 47 patients who underwent preoperative esophagoscopic biopsy, only 1 patient was diagnosed with EMEC. EMEC was more often found in female patients (39.7% versus 25.8%, P=0.036) and patients with EMEC had a significantly lower rate of lymph node metastasis (25.0% versus 49.4%, P<0.001) than patients with ESCC. After 1:1 propensity score matching, the 5-year overall survival rate of 55.2% for patients with EMEC was similar to that of 61.9% for patients with ESCC (P=0.399).

**Conclusions:**

EMEC is a rare disease that more often affects females and these patients has less lymph node metastasis than patients with ESCC. Preoperative esophagoscopic biopsy has difficulty obtaining an accurate pathological diagnosis for EMEC patients. The prognosis for EMEC is similar to that for ESCC.

## Introduction

Esophageal mucoepidermoid carcinoma (EMEC), which is characterized by a mixture of squamous, mucinous and intermediate components (1, 2), is very rare, with an incidence of less than 1% of all esophageal carcinomas (3). Most of the previous studies on EMEC were case reports (4–21), and only six series with a few cases have been reported to date (3, 22–26). The biological behavior and treatment of this rare malignancy are not well established.

In a previous study (22), we reported data from 36 patients with EMEC, which was the largest series to date, and found that EMEC was easily misdiagnosed by endoscopic biopsy and these patients had poorer survival than patients with esophageal squamous cell carcinoma (ESCC). As the patient number was still very small and no detailed analyses between EMEC and ESCC were conducted, we think it is necessary to perform further study to give us a better understanding of this rare disease. In this study, we reviewed data from 58 patients with EMEC and aimed to investigate its biological behavior and treatment. We further used the propensity score matching (PSM) analysis to match the baseline between patients with EMEC and ESCC to compare their prognosis.

## Patients and Methods

Five thousand eight hundred eighty-one patients with esophageal carcinoma underwent esophagectomy with lymphadenectomy in the Department of Thoracic Surgery, Shantou University Medical College Cancer Hospital between January 1995 and December 2019. Fifty-eight patients (0.99%, 58/5881) were histopathologically diagnosed with EMEC and enrolled in the current study. All patients provided informed consent. This study was approved by an independent ethics committee at our hospital.

### Data Collection

All clinicopathological data and laboratory data were retrospectively investigated. All specimens were re-examined by an expert pathologist (Dr. Xiao-long Wei). Tumors were graded according to the Armed Forces Institute of Pathology (AFIP) criteria (27). In briefly, five histopathologic features that indicated high grade behavior were set individual point values (**Table 1**). A quantitative grading system based on a point score for each of the five histopathologic features was employed. Tumors were differentiated into low grade (0-4 points), intermediate (5-6 points), and high grade (7-14 points). Tumors were staged according to the 8th edition of the AJCC TNM staging system.

**Table 1:** Grading parameters and point values for esophageal mucoepidermoid carcinoma.

Parameters	Points
Intracystic component <20%	2
Neural invasion present	2
Necrosis present	3
Mitosis (4 or more per 10 high-power fields)	3
Anaplasia present	4

### Treatment

A left thoracotomy was routinely conducted for the patients who underwent esophagectomy before 2010, and a right thoracotomy was conducted for most of the patients after 2011. For lymphadenectomy, the paraesophageal, subcarinal, supradiaphragmatic, paracardial, lesser curvature, and left gastric lymph nodes were routinely dissected for all patients. The lymph nodes around the left and right recurrent nerves and the common hepatic lymph nodes were also resected for patients who underwent a right thoracotomy.

None of these patients received preoperative neoadjuvant therapy. Postoperative adjuvant therapy was recommended to patients with locally advanced diseases in our hospital. A total of 18 patients received postoperative adjuvant therapy, including 15 patients who received adjuvant radiotherapy, 3 patients who received adjuvant chemotherapy, and 1 patient who received adjuvant chemoradiotherapy. The regimen for chemotherapy was paclitaxel + cisplatin. The total dose for adjuvant radiotherapy was 44-60 Gy (median 50 Gy).

### Statistical Analysis

Statistical analysis was performed using SPSS 26.0 software (IBM, Armonk, New York, USA) and Jamovi version 2.2.5. Categorical variables were compared by Pearson’s chi-square test or Fisher’s exact test. Overall survival (OS) was calculated by the Kaplan–Meier method, and the log-rank test was used to assess the survival differences. Multivariate regression analysis was applied to determine the independent prognostic factors by the Cox proportional-hazards model. PSM was conducted with the 1:1 nearest neighbor matching method. The covariates included sex, age, tumor location, tumor length, histologic grade, thoracotomy, resection margin, pT category, pN category, and adjuvant radiotherapy. P< 0.05 was set to indicate significance.

## Results

### Clinicopathological Features of the Patients With EMEC

Of the 58 cases with EMEC, 57 cases were pure type of mucoepidermoid carcinomas, while the other one case with co-occurrence of ESCC and EMEC. The study cohort included 36 males and 22 females with a median age of 59 years (range, 40-78 years). Most of the tumors (67.2%, 39/58) were located on the middle third of the thoracic esophagus, and the median length was 5.0 cm (range, 1.5-8.0 cm). Based on the AFIP criteria, this study included 17 patients with low-grade tumors (Grade 1), 28 patients with intermediate-grade tumors (Grade 2), and 13 patients with high-grade tumors (Grade 3). Forty-seven patients underwent preoperative esophagoscopy with biopsy, while the other 11 patients in the earlier years did not receive esophagoscopy. Only 1 patient was diagnosed with EMEC in preoperative esophagoscopic biopsy. Forty-three patients were misdiagnosed with ESCC, two patients were misdiagnosed with esophageal adenosquamous carcinoma, and one patient was misdiagnosed with esophageal adenocarcinoma.

Based on the 8th edition of the AJCC TNM staging system, there was 1 case of pT1 disease, 10 cases of pT2 disease, 36 cases of pT3 disease, and 11 cases of pT4 disease. A total of 1039 lymph nodes were resected, while 44 nodes were pathologically diagnosed as metastatic. The mean number of resected lymph nodes was 17.9 ± 1.2, with a median number of 16 (range, 4–46). There were 43 cases of pN0 disease, 10 cases of pN1 disease, 3 cases of pN2 disease, and 2 cases of pN3 disease.

Six patients suffered major postoperative complications, including 3 cases of pneumonia, 2 cases of esophagogastric anastomotic leaks, and 1 case of chylothorax. No patient died during treatment in the hospital.

### Comparison of Clinicopathological Features Between the Patients With EMEC and ESCC

Of the 5881 patients with esophageal cancer who underwent esophagectomy in our hospital between January 1995 and December 2019, 5558 patients were histopathologically diagnosed with ESCC. We excluded 477 patients who received neoadjuvant therapy (including 256 patients who received neoadjuvant chemoradiotherapy, 191 patients who received neoadjuvant radiotherapy, and 30 patients who received neoadjuvant chemotherapy), and 53 patients lacked any follow-up data, leaving a cohort of 5028 patients with ESCC for analysis.

The clinicopathological features between the patients with EMEC and ESCC are shown in **Table 2**. All of the factors were balanced between the patients with EMEC and ESCC, except for sex and the pN category. EMEC was found more often in female patients than ESCC (39.7% versus 25.8%, P=0.036). Moreover, the patients with EMEC had a significantly lower rate of lymph node metastasis than the patients with ESCC (25.0% versus 49.4%, P<0.001).

**Table 2:** Clinicopathological features between patients with EMEC and ESCC in the original cohort and matched cohort.

Variable	Original cohort		*P* value	Matched cohort		*P* value
	EMEC (n = 58)	ESCC (n = 5028)		EMEC (n = 58)	ESCC (n = 58)	
Sex			0.036			0.849
Male	36 (62.1%)	3731 (74.2%)		36 (62.1%)	35 (60.3%)	
Female	22 (37.9%)	1297 (25.8%)		22 (37.9%)	23 (39.7%)	
Age (yr)			0.381			1.000
≤60	32 (55.2%)	3058 (60.8%)		32 (55.2%)	32 (55.2%)	
>60	26 (44.8%)	1970 (39.2%)		26 (44.8%)	26 (44.8%)	
Tumor location			0.638			0.676
Upper third	10 (17.2%)	655 (13.0%)		10 (17.2%)	7 (12.1%)	
Middle third	39 (67.2%)	3544 (70.5%)		39 (67.2%)	43 (74.1%)	
Lower third	9 (15.2%)	829 (16.5%)		9 (15.2%)	8 (13.8%)	
Tumor length			0.668			0.699
≤4cm	22 (37.9%)	1771 (35.2%)		22 (37.9%)	20 (34.5%)	
>4cm	36 (62.1%)	3257 (64.8%)		36 (62.1%)	38 (65.5%)	
Histologic grade			0.189			0.908
Grade 1	17 (29.3%)	1520 (30.2%)		17 (29.3%)	15 (30.2%)	
Grade 2	28 (48.3%)	2797 (55.6%)		28 (48.3%)	30 (55.6%)	
Grade 3	13 (22.4%)	711 (14.1%)		13 (22.4%)	13 (14.1%)	
Thoracotomy			0.315			0.842
Left thoracotomy	40 (69.0%)	3758 (74.7%)		40 (69.0%)	39 (67.2%)	
Right thoracotomy	18 (31.0%)	1270 (25.3%)		18 (31.0%)	19 (32.8%)	
Resection margin			0.636			0.697
Radical	54 (93.1%)	4593 (91.3%)		54 (93.1%)	55 (94.8%)	
Palliative	4 (6.9%)	435 (8.7%)		4 (6.9%)	3 (5.2%)	
pT category			0.239			1.000
pT1-T2	11 (19.0%)	1295 (25.8%)		11 (19.0%)	11 (19.0%)	
pT3-T4	47 (81.0%)	3733 (74.2%)		47 (81.0%)	47 (81.0%)	
pN category			<0.001			0.830
pN0	43 (74.1%)	2543 (50.6%)		43 (74.1%)	44 (75.9%)	
pN1-N3	15 (25.9%)	2485 (49.4%)		15 (25.9%)	14 (24.1%)	
Ajuvant radiotherapy			0.375			0.672
Yes	16 (27.6%)	1140 (22.7%)		16 (27.6%)	14 (24.1%)	
No	42 (72.4%)	3888 (77.3%)		42 (72.4%)	44 (75.9%)	

EMEC, esophageal mucoepidermoid carcinoma; ESCC, esophageal squamous cell carcinoma.

### Prognosis and Survival Analysis for the Patients With EMEC

Follow-up was continued to December 2020. Twenty-nine patients died during the follow-up period.

**Figure 1:**
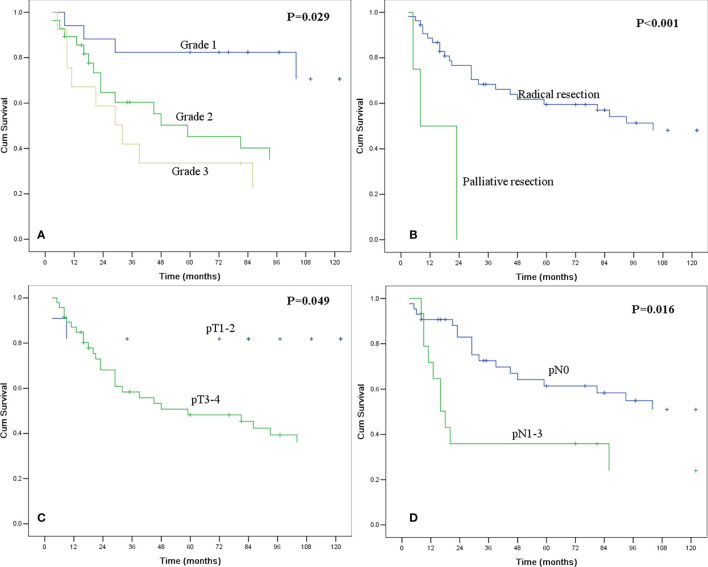
Kaplan-Meier curves for overall survival according to histologic grade **(A)**, resection margin **(B)**, pT category **(C)**, and pN category **(D)**. All of the survival differences were significant (P <0.05).

The median survival time (MST) for the entire group was 93.0 months (95% confidence interval [CI] 33.2-152.8), and the 1-, 3- and 5-year OS rates were 86.0%, 63.3% and 55.2%, respectively. In the univariate analysis (**Table 3**), the histological grade, resection margin, pT category, and pN category were significantly correlated with survival (**Figure 1**). These four factors were enrolled in a multivariate analysis. However, only the resection margin and pN category were found to be independent predictors (**Table 4**). The histological grade and pT category were not independent prognostic factors.

**Table 3:** Univariate analysis in regard to overall survival according to clinicopathological features for 58 patients with EMEC.

Variable	No. of patients	1-yr OS (%)	3-yr OS (%)	5-yr OS (%)	Hazard Ratio	*P* value
Sex					1.168	0.614
Male	36	86.0	64.1	57.4		
Female	22	85.9	62.0	51.7		
Age (yr)					1.463	0.312
≤60	32	87.5	62.5	59.0		
>60	26	84.2	64.8	49.9		
Tumor location					1.010	0.918
Upper third	10	80.0	60.0	50.0		
Middle third	39	86.9	63.2	56.9		
Lower third	9	88.9	66.7	53.3		
Tumor length					1.202	0.487
≤4cm	22	90.9	66.7	61.2		
>4cm	36	82.8	61.3	51.6		
Histologic grade					2.058	0.029
Grade 1	17	94.1	82.4	82.4		
Grade 2	28	89.3	60.3	45.3		
Grade 3	13	67.1	42.0	33.6		
Thoracotomy					1.009	0.908
Left thoracotomy	40	87.5	62.5	54.7		
Right thoracotomy	18	81.9	66.7	57.2		
Resection margin					7.089	<0.001
Radical	54	88.7	68.3	59.5		
Palliative	4	50.0	0	0		
pT category					4.151	0.049
pT1-2	11	81.8	81.8	81.8		
pT3-4	47	87.0	58.4	48.2		
pN category					2.516	0.016
pN0	43	90.7	72.6	61.4		
pN1-3	15	71.8	35.9	35.9		
Adjuvant radiotherapy					0.695	0.401
Yes	16	93.8	62.5	62.5		
No	42	83.3	63.4	52.3		

OS, overall survival.

**Table 4:** Multivariate analysis in regard to overall survival for 58 patients with EMEC.

Prognostic factor	Hazard Ratio	95%CI	*P* value
Histologic grade	1.458	0.816-2.603	0.203
Resection margin	7.122	2.202-23.036	0.001
pT category	4.254	0.923-19.610	0.063
pN category	3.230	1.303-8.006	0.011

CI, confidence interval.

### Comparison of Survival Between the Patients With EMEC and ESCC

**Figure 2:**
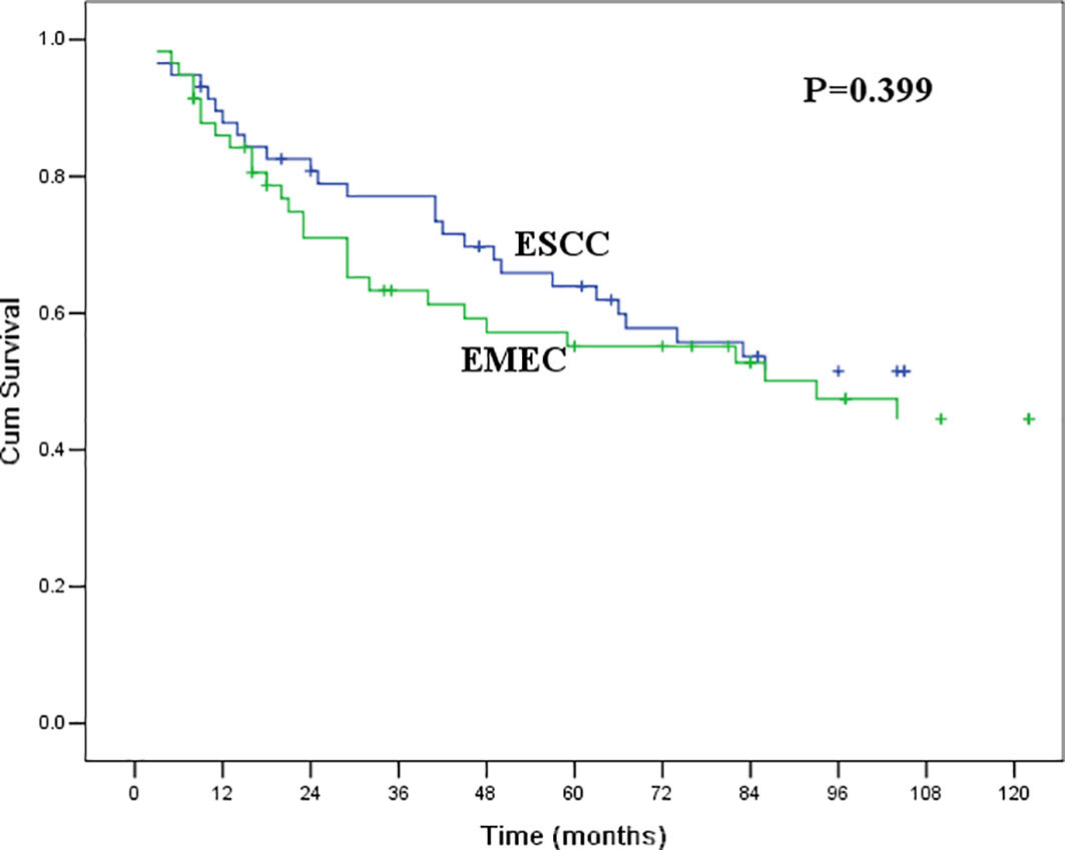
Kaplan-Meier curves for overall survival between patients with ESCC and EMEC. The survival difference was not significant (P=0.399).

As the baseline clinicopathological features of patients with EMEC and ESCC in the original cohort were not comparable in this study, we used PSM analysis to match the characteristics of patients with EMEC and ESCC before survival analysis. After 1:1 propensity score matching, there were no significant differences in clinicopathological features between patients with EMEC and ESCC (**Table 2**). The 5-year overall survival rate was 61.9% for patients with ESCC, which was similar to that of 55.2% for patients with EMEC (P=0.399, **Figure 2**).

## Discussion

EMEC is a very rare disease and accounts for less than 1% of all cases of primary esophageal cancer (3). To date, only approximately 100 cases of this disease have been reported in the English literature (3–26). In a previous study (22), we retrospectively analyzed the clinical features and prognosis of 36 patients with EMEC, which is the largest series to date, and compared their prognoses with those of patients with ESCC. However, as the patient number was still very small and some of the clinical data and follow-up data for the patients with ESCC in the early period (before 1995) were lacking, we could not conduct a detailed analysis to compare the clinical features between EMEC and ESCC patients, and the survival comparison might not be accurate. Therefore, we think it is necessary to conduct further studies to provide a better understanding of this rare disease.

In the current study, we included only patients who underwent esophagectomy after 1995 and excluded all the data in the early period (before 1995). Finally, six patients with EMEC in the early period were excluded, and a total of 58 patients were enrolled in this study, which accounted for 0.99% (58/5881) of all esophageal carcinoma patients who underwent esophagectomy at the same time. The median age for all patients was 59 years, and most of the lesions (67.2%, 39/58) were located at the middle third of the thoracic esophagus, which was similar to those of the patients with ESCC. However, it seemed that EMEC was found more often in female patients than ESCC (male:female ratio of 3:2 in the EMEC group versus that of 3:1 in the ESCC group, P=0.036). Due to the low incidence of EMEC and the small patient numbers in previous studies, it is difficult to investigate the morbidity difference in sex between EMEC and ESCC. Kumagai et al. (28) conducted a literature review and enrolled 125 patients with EMEC from 56 English and Japanese articles. They found that the male:female ratio was 3.2:1, which was lower than that of the conventional male:female ratio of 6.5:1 for ESCC in Japan. However, as not all of the patients enrolled in this study were Japanese, it was not reasonable to compare the sex ratio with that of ESCC patients in Japan. All of the patients with EMEC and ESCC enrolled in this study were treated in a single center at the same time, and we think that our results might be more convincing.

Due to the small volume of biopsy specimens from esophagoscopies, it is difficult to obtain an accurate pathological diagnosis before surgery, as the glandular component might not be apparent. In the review by Kumagai et al. (28), only 14.3% of the patients were diagnosed with EMEC by endoscopic biopsy, and most of them (77.1%) were misdiagnosed with ESCC. In the current study, of the 47 patients who received preoperative esophagoscopy with biopsy, only 1 patient (2.1%) was diagnosed with EMEC by biopsy, while most of them (91.5%) were misdiagnosed with ESCC. The diagnosis of these carcinomas often requires resection specimens (29).

In our current study, 25.9% of the patients with EMEC were confirmed to have lymph node metastasis by postoperative pathological examination, which was significantly lower than the 49.4% of patients with ESCC confirmed to have metastasis. No previous studies have compared the difference in lymph node metastasis between patients with EMEC and ESCC. In the review by Kumagai et al. (28), 48.9% (45/92) of the patients with EMEC were proven to have lymph node metastasis. However, as the studies enrolled in this review were from various hospitals and countries over a long period, we were unable to compare the rate of lymph node metastasis of patients with EMEC with that of patients with ESCC. As most of the other clinicopathological features were comparable between the patients with EMEC and ESCC in this study, especially for the thoracotomy and pT category, we think our finding that patients with EMEC have a lower rate of lymph node metastasis than patients with ESCC is still of clinical significance. However, the reason why patients with EMEC have less lymph node metastasis is not clear, and we think further study should be conducted to investigate this.

Esophagectomy with lymphadenectomy is still the most important treatment for esophageal carcinoma, while neoadjuvant chemoradiotherapy is recommended for locally advanced disease (30). As the incidence of EMEC is too low and most cases of this disease are misdiagnosed by preoperative esophagoscopic biopsy, it is difficult to investigate the value of neoadjuvant chemoradiotherapy for this disease. All of our patients received surgical resection as their initial treatment, and 18 patients received postoperative adjuvant therapy. Although the 5-year OS of 62.5% for patients who received adjuvant therapy was higher than that of 52.3% for patients who did not receive adjuvant therapy, the difference was not significant. As the patient number is still too small, we think further studies are still needed to evaluate the value of postoperative adjuvant therapy for patients with EMEC.

Because of the rarity of EMEC, the prognosis of this disease has not been well established. Although some of the previous studies found that EMEC might be more aggressive than ESCC (3, 6, 13), other studies obtained different results (18, 26). In our previous study (22), we found that the 5-year OS rate of 25.8% for patients with EMEC was lower than the 5-year absolute survival rate of 39.2% for patients with ESCC. However, we did not obtain a consistent result in this study. In the current study, after propensity score matching, we found that the survival for patients with EMEC was similar to that for patients with ESCC (P=0.399). The prognosis for patients with ESCC and EMEC has improved over the past decade (28, 30). As follow-up data for many of the patients with ESCC in the early period (before 1995) were lacking, the survival rate for patients with ESCC in our previous study might be overevaluated. Moreover, we did not conduct PSM analysis to match the baseline of the clinicopathological features between patients with EMEC and ESCC in our previous study. According to our current findings, we think that EMEC may not be more aggressive than ESCC, and the resection margin and pN category were independent predictors for this disease.

Our study still has some limitations. First, although we report the largest ever series in this study, the patient number may still not be large enough to evaluate some of the other independent prognostic factors, such as the pT category and histological grade. Second, many other prognostic factors, such as the lymphovascular invasion, has been known to be a poor prognostic factor for many cancers. However, we did not recorded the information of these factors in this study (31). Third, we could not promote a multidisciplinary treatment strategy for EMEC in this study, especially for advanced disease. However, as there are few studies concerned with this rare disease to date, we think that our current study still provides a better understanding of EMEC.

In conclusion, EMEC is a rare disease and may more often affect females and have less lymph node metastasis than ESCC. Preoperative esophagoscopic biopsy has difficulty obtaining an accurate pathological diagnosis for EMEC. The prognosis for patients with EMEC is not poorer than that for patients with ESCC, and the resection margin and pN category are independent predictors. Further studies are needed to evaluate our findings and investigate a multidisciplinary treatment strategy for EMEC.

## Author Contributions

S-BC designed the research and wrote part of the paper. XW analyzed the data and wrote part of the paper. Y-PC wrote part of the paper. All authors contributed to the article and approved the submitted version.

## Funding

Science and Technology Special Fund of Guangdong Province of China(190829105556145), The Medical Scientific Research Foundation of Guangdong Province of China (B2019070).

## Conflict of Interest

The authors declare that the research was conducted in the absence of any commercial or financial relationships that could be construed as a potential conflict of interest.

## Publisher’s Note

All claims expressed in this article are solely those of the authors and do not necessarily represent those of their affiliated organizations, or those of the publisher, the editors and the reviewers. Any product that may be evaluated in this article, or claim that may be made by its manufacturer, is not guaranteed or endorsed by the publisher.

## References

[1] Lam AK. Histopathological Assessment for Esophageal Adenocarcinoma. Methods Mol Biol (2018) 1756:67–76. doi: 10.1007/978-1-4939-7734-5_6 29600360

[2] Lam AK Ochiai A Odze RD. Tumours of the Oesophagus: Introduction. In: Odze RD Lam AK Ochiai A Washington MK, editors. WHO Classification of Tumours-Digestive System Tumours, 5th. Lyon: IARC (2019). p. 28–9. Chapter 2.

[3] Hagiwara N Tajiri T Tajiri T Miyashita M Sasajima K Makino H. Biological Behavior of Mucoepidermoid Carcinoma of the Esophagus. J Nippon Med Sch (2003) 70(5):401–7. doi: 10.1272/jnms.70.401 14578940

[4] Liu C Zhao Y Chu W Zhang F Zhang Z. Mucoepidermoid Carcinoma of Esophagus Combined With Squamous Carcinoma of Lung: A Case Report and Literature Review. J Cancer Res Ther (2015) 11(3):658. doi: 10.4103/0973-1482.140986 26458663

[5] Kiyozaki H Obitsu T Ishioka D Saito M Chiba F Takata O. A Rare Case of Primary Mucoepidermoid Carcinoma of the Esophagus. Clin J Gastroenterol (2015) 8(1):26–8. doi: 10.1007/s12328-014-0546-7 25475139

[6] Koide N Hamanaka K Igarashi J Hanazaki K Adachi W Hosaka S. Co-Occurrence of Mucoepidermoid Carcinoma and Squamous Cell Carcinoma of the Esophagus: Report of a Case. Surg Today (2000) 30(7):636–42. doi: 10.1007/s005950070104 10930230

[7] Sasajima K Watanabe M Takubo K Takai A Yamashita K Onda M. Mucoepidermoid Carcinoma of the Esophagus: Report of Two Cases and Review of the Literature. Endoscopy (1990) 22(3):140–3. doi: 10.1055/s-2007-1012820 2192858

[8] Zheng C Chen X Zhang F Yan L Zhang X. Surgery Combined With Radio-Chemotherapy for Esophageal Mucoepidermoid Carcinoma: A Case Report. Med (Baltimore) (2018) 97(24):e11165. doi: 10.1097/MD.0000000000011165 PMC6023669 29901650

[9] Matsufuji H Kuwano H Ueo H Sugimachi K Inokuchi K. Mucoepidermoid Carcinoma of the Esophagus–A Case Report. Jpn J Surg (1985) 15(1):55–9. doi: 10.1007/BF02469858 3990050

[10] Woodard BH Shelburne JD Vollmer RT Postlethwait RW. Mucoepidermoid Carcinoma of the Esophagus: A Case Report. Hum Pathol (1978) 9(3):352–4. doi: 10.1016/s0046-8177(78)80093-8 658967

[11] Mewa Kinoo S Maharaj K Singh B Govender M Ramdial PK. Primary Esophageal Sclerosing Mucoepidermoid Carcinoma With “Tissue Eosinophilia”. World J Gastroenterol (2014) 20(22):7055–60. doi: 10.3748/wjg.v20.i22.7055 PMC4051951 24944502

[12] Liu ZJ Sun SY Guo JT Wang S Ge N Liu X. A Primary Esophageal Mucoepidermoid Carcinoma Mimicking a Benign Submucosal Tumor. Dis Esophagus (2012) 25(2):178–9. doi: 10.1111/j.1442-2050.2010.01158.x 22335203

[13] Turkyilmaz A Eroglu A Gursan N. Muco-Epidermoid Carcinoma of the Oesophagus: A Case Report. Acta Chir Belg (2009) 109(3):416–8. doi: 10.1080/00015458.2009.11680452 19943606

[14] Tamura S Kobayashi K Seki Y Matsuyama J Kagara N Ukei T. Mucoepidermoid Carcinoma of the Esophagus Treated by Endoscopic Mucosal Resection. Dis Esophagus (2003) 16(3):265–7. doi: 10.1046/j.1442-2050.2003.00342.x 14641323

[15] Mafune K Takubo K Tanaka Y Fujita K. Sclerosing Mucoepidermoid Carcinoma of the Esophagus With Intraepithelial Carcinoma or Dysplastic Epithelium. J Surg Oncol (1995) 58(3):184–90. doi: 10.1002/jso.2930580309 7898115

[16] Ozawa S Ando N Shinozawa Y Ohmori T Kase K Sato T. Two Cases of Resected Esophageal Mucoepidermoid Carcinoma. Jpn J Surg (1989) 19(1):86–92. doi: 10.1007/BF02471574 2733284

[17] Pascal RR Clearfield HR. Mucoepidermoid (Adenosquamous) Carcinoma Arising in Barrett’s Esophagus. Dig Dis Sci (1987) 32(4):428–32. doi: 10.1007/BF01296298 3549205

[18] Bell-Thomson J Haggitt RC Ellis FH Jr. Mucoepidermoid and Adenoid Cystic Carcinomas of the Esophagus. J Thorac Cardiovasc Surg (1980) 79(3):438–46. doi: 10.1016/0022-2828(80)90132-7 6243727

[19] Osamura RY Sato S Miwa M Miwa T. Mucoepidermoid Carcinoma of the Esophagus. Report of an Unoperated Autopsy Case and Review of Literature. Am J Gastroenterol (1978) 69(4):467–70. 685954

[20] Weitzner S. Mucoepidermoid Carcinoma of Esophagus. Report of a Case. Arch Pathol (1970) 90(3):271–3. 5451894

[21] Kay S. Mucoepidermoid Carcinoma of the Esophagus. Report of Two Cases. Cancer (1968) 22(5):1053–9. doi: 10.1002/1097-0142(196811)22:5<1053::aid-cncr2820220522>3.0.co;2-s 5686635

[22] Chen S Chen Y Yang J Yang W Weng H Li H. Primary Mucoepidermoid Carcinoma of the Esophagus. J Thorac Oncol (2011) 6(8):1426–31. doi: 10.1097/JTO.0b013e31821cfb96 21587086

[23] Lieberman MD Franceschi D Marsan B Burt M. Esophageal Carcinoma. The Unusual Variants. J Thorac Cardiovasc Surg (1994) 108(6):1138–46. doi: 10.1016/S0022-5223(94)70158-X 7983884

[24] Lam KY Dickens P Loke SL Fok M Ma L Wong J. Squamous Cell Carcinoma of the Oesophagus With Mucin-Secreting Component (Muco-Epidermoid Carcinoma and Adenosquamous Carcinoma): A Clinicopathologic Study and a Review of Literature. Eur J Surg Oncol (1994) 20(1):25–31. 8131864

[25] Lam KY Loke SL Ma LT. Histochemistry of Mucin Secreting Components in Mucoepidermoid and Adenosquamous Carcinoma of the Oesophagus. J Clin Pathol (1993) 46(11):1011–5. doi: 10.1136/jcp.46.11.1011 PMC501684 7504701

[26] Fegelman E Law SY Fok M Lam KY Loke SL Ma LT. Squamous Cell Carcinoma of the Esophagus With Mucin-Secreting Component. Mucoepidermoid Carcinoma J Thorac Cardiovasc Surg (1994) 107(1):62–7. doi: 10.1016/S0022-5223(94)70454-6 8283920

[27] Goode RK Auclair PL Ellis GL. Mucoepidermoid Carcinoma of the Major Salivary Glands: Clinical and Histopathologic Analysis of 234 Cases With Evaluation of Grading Criteria. Cancer (1998) 82(7):1217–24. doi: 10.1002/(sici)1097-0142(19980401)82:7<1217::aid-cncr2>3.0.co;2-c 9529011

[28] Kumagai Y Ishiguro T Kuwabara K Sobajima J Fukuchi M Ishibashi K. Primary Mucoepidermoid Carcinoma of the Esophagus: Review of the Literature. Esophagus (2014) 11:81–8. doi: 10.1007/s10388-014-0414-z

[29] Lam AK. Updates on World Health Organization Classification and Staging of Esophageal Tumors: Implications for Future Clinical Practice. Hum Pathol (2021) 108:100–12. doi: 10.1016/j.humpath.2020.10.015 33157124

[30] Rustgi AK El-Serag HB. Esophageal Carcinoma. N Engl J Med (2014) 371(26):2499–509. doi: 10.1056/NEJMra1314530 25539106

[31] Okubo Y Sato S Osaka K Yamamoto Y Suzuki T Ida A. Clinicopathological Analysis of the ISUP Grade Group And Other Parameters in Prostate Cancer: Elucidation of Mutual Impact of the Various Parameters. Front Oncol (2021) 11:695251. doi: 10.3389/fonc.2021.695251 34395260 PMC8356042

